# Smartphone‐based augmented reality physiology and anatomy laboratories

**DOI:** 10.1111/medu.14756

**Published:** 2022-02-21

**Authors:** Christian Moro, Charlotte Phelps

## 1 WHAT PROBLEMS WERE ADDRESSED?

With a developing focus towards the online provision of education, it has become increasingly challenging to run regular face‐to‐face laboratories within medical and health science programmes. In disciplines such as physiology and anatomy, silicone models are often used to exhibit the essential features of organs and their three‐dimensional (3‐D) interrelationships with nearby structures. This is core to the learning required, as these concepts are not easily visualised from two‐dimensional illustrations, photographs, videos or teaching slides. Augmented reality, running on the students' own smartphones, uses the device's camera to incorporate a 3‐D model onto the real environment surrounding the learner.^1^ In addition, augmented reality models can be animated to show the movements of organs (e.g., the heart beating), progression of diseases (e.g., lesions forming in multiple sclerosis), or other considerations important to the lesson. In this way, augmented reality can bridge the gap between the benefits usually obtained from face‐to‐face laboratories and the inhibitions when attempting to learn these concepts remotely.

## 2 WHAT WAS TRIED?

In classes that were formerly run as face‐to‐face laboratories with silicone or physical models, an augmented reality application was created in‐house (using Unity3D, C# coding and Cinema4D) and exported to a downloadable format (Android devices) or website (all other devices). Alternatively, it should be noted that there are several free augmented reality‐enabled apps available online (e.g., visiblebody.com), making this technology available to educators in many cases. While watching the lessons online, students used their smartphones and pointed the camera at a marker provided at selected spots throughout the teaching slides. As the educator described the model and features, users could enlarge the visualisation by moving the phone closer to the marker. Tapping on the screen highlighted parts of the model and presented the region's names, with a ‘dissect’ button available to remove layers and view underlying features. An undo command allowed users to revert changes, but in most cases, learners were free to explore the model at their own will while it was being described by the educator.

## 3 WHAT LESSONS WERE LEARNED?

Through smartphone‐based augmented reality, online students were able to benefit from viewing 3‐D models wherever they were in the world. This provided a much more interactive and enhanced learning experience during online teaching sessions, compared to following along solely with pictures and illustrations presented on a slideshow. After the session, student comments on written evaluations included: ‘this is good because I can visually see it while it was being talked about’; ‘this is making it easy to understand’; ‘very cool to have a visual format in addition to lecture‐style learning’; and ‘this allows a good level of engagement with minimal distractions’. Due to the widespread availability of smartphones, no student had any issues accessing the application. In cases where it is not possible to have on‐campus laboratories and lectures (or when instructing through a multi‐modal environment), this form of modern technology can empower educators to provide innovative, exciting, self‐directed, and technology‐enhanced learning opportunities for students.
